# Selection of suitable housekeeping genes for gene expression analysis in glioma using quantitative real-time PCR

**DOI:** 10.1186/1755-8166-7-S1-P18

**Published:** 2014-01-21

**Authors:** Madhuri GS Aithal, Narayanappa Rajeswari

**Affiliations:** 1 Department of Biotechnology, Dayananda Sagar College of Engineering, Bangalore, Karnataka, India- 560078

## Background

Quantitative real-time PCR (qPCR) is the most reliable tool for gene expression measurements from tissue samples. Selection of housekeeping genes (HKGs) that are most stably expressed among tissues is vital to carry out accurate gene expression profiling of target genes. Expression of HKGs varies among tissues and experimental conditions. There is no 'universal' housekeeping gene having stable expression in all tissues under all experimental conditions. So, it is extremely important to identify most appropriate internal control genes for a particular tissue and experimental conditions. The aim of the present study is to identify most suitable HKGs for gene expression analysis in glioma tissue samples.

## Material and methods

Based on literature survey six most commonly used HKGs reported to be invariant in human gliomas were chosen for gene expression analysis. We performed qPCR using RNA from formalin fixed paraffin embedded glioma patient samples and normal brain samples to investigate the expression pattern of six HKGs [Glyceraldehyde-3-phosphate dehydrogenase (GAPDH), hypoxanthine phosphoribosyltransferase 1 (HPRT), β2 microglobulin (B2M), TATA-binding protein (TBP), 18S ribosomal RNA (RN18S1) and ribosomal protein L13a (RPL13A)] with different abundance. A simple ΔCt approach was employed to calculate the fold change.

## Results and conclusions

Our data showed that out of the six genes studied, expression of GAPDH, B2M and RPL13A were found to have most constant expression across all the tumor samples in our experimental setup. Thus, these three genes proved to be the most suitable to be used as internal controls for gene expression analysis in human glioma. Except for GAPDH, none of the other conventionally used HKGs in glioma studies e.g., HPRT and TBP were found to be suitable as they showed large variation in RNA expression. Validation of housekeeping genes is therefore highly specific for a particular experimental setup and is important in assessing any new setup.

